# Glia in Neurodegeneration: The Housekeeper, the Defender and the Perpetrator

**DOI:** 10.3390/ijms21239188

**Published:** 2020-12-02

**Authors:** Carrie Sheeler, Juao-Guilherme Rosa, Austin Ferro, Brian McAdams, Ella Borgenheimer, Marija Cvetanovic

**Affiliations:** 1 Department of Neuroscience, University of Minnesota, Minneapolis, MN 55455, USA; jrosa@umn.edu (J.-G.R.); ferro029@umn.edu (A.F.); mcada006@umn.edu (B.M.); borge248@umn.edu (E.B.); mcvetano@umn.edu (M.C.); 2 Institute for Translational Neuroscience, University of Minnesota, Minneapolis, MN 55455, USA

**Keywords:** astrocytes, microglia, neurodegeneration, mouse, human

## Abstract

Over the past decade, research has unveiled the intimate relationship between neuroinflammation and neurodegeneration. Microglia and astrocytes react to brain insult by setting up a multimodal inflammatory state and act as the primary defenders and executioners of neuroinflammatory structural and functional changes. Microglia and astrocytes also play critical roles in the maintenance of normal brain function. This intricate balance of homeostatic and neuroinflammatory functions can influence the onset and the course of neurodegenerative diseases. The emergent role of the microglial-astrocytic axis in neurodegenerative disease presents many druggable targets that may have broad therapeutic benefits across neurodegenerative disease. Here, we provide a brief review of the basal function of both microglia and astrocytes, how they are changed in disease states, the significant differences between mouse and human glia, and use of human induced pluripotent stem cells derived from patients to study cell autonomous changes in human astrocytes and microglia.

## 1. Physiological Roles of Astrocytes and Microglia

Much understanding of the roles of glial cells in disease can be derived from knowing their origins and their normal function.

Unlike other brain cells, microglia originate from the erythromyeloid progenitors that in mice develop around embryonic day 8.5 (E8.5) in the yolk sack. Erythromyeloid progenitors migrate to the brain starting around E9.5 and continue to do so until the blood–brain barrier is formed around E14 [1,2]. During early development, microglia mature in concert with local neuronal populations and have lasting effects on the developing neural architecture, including their well-understood role in removing superfluous neurons and synaptic pruning [3,4]. Consequently, abnormalities in developmental functions of microglia may cause sculpting of the brain network that is more sensitive to injuries during adulthood [5].

In the adult brain, in order to maintain homeostasis, microglia constantly extend and retract their processes to survey the local environment for synaptic activity [6], pathogen presence, and injury [7,8]. It was recently demonstrated that this constant surveillance by microglia depends, in part, on the expression of the tandem pore domain halothane-inhibited potassium channel (THIK1) [8]. It was shown that tonic THIK1 activity maintained microglial membrane potential, and blocking THIK1 caused reduction of microglial ramification and surveillance [8]. These results implicate a potential role of extracellular potassium concentration, which is regulated by local neuronal and astrocytic activity, as a control mechanism for microglial surveillance. While it is unclear whether physiological changes in extracellular potassium concentration are sufficiently large to alter microglial surveillance, pathological changes in neuronal or astroglial function are likely to do so. For example, it is possible that in disease conditions enhanced neuronal activity [9] and/or reduced clearance by astrocytes may increase extracellular potassium [10]. Such changes in the local cellular environment could alter THIK1 activity and consequently microglial surveillance and morphology.

Age as a risk factor and selective brain region vulnerabilities characterize many neurodegenerative diseases. Increasing evidence of microglial regional heterogeneity and changes with age further promote the concept that physiological characteristics of glia can contribute to aspects of disease pathogenesis. Indeed, several studies to date have provided evidence of spatial and temporal microglial heterogeneity under steady-state conditions [11,12]. For example, cerebellar microglia display morphological features and gene expression indicating enhanced clearance functions, making them notably distinct from striatal microglia [11]. Understanding the regional characteristics of microglia and how aging effects them is important for determining their function both in healthy and aging brains but also unearthing their possible contribution to regional susceptibility in neurological diseases. It is possible that the intimate connection between neurons and microglia contributes to the regional and temporal heterogeneity of microglia, as there is an association between neurons and the increased clearance by cerebellar microglia [11].

In contrast to microglia, astrocytes arise from ectoderm through mostly symmetrical division (post-natal) and to a lesser extent asymmetrical division (early embryogenesis) of radial glial stem cells and their differentiation into intermediate glial progenitor cells. As they develop, astroglia take on unique morphologies that are as varied across anatomical regions of the brain as are neuronal differences [13]. Similar to microglia, astrocytic transcriptomes are distinct between and within brain regions [14,15,16]. These regional differences in transcriptomes are thought to be due to both the different neural environments during development [17], as well as the regional differences in secreted factors such as sonic hedgehog [14].

Even with these regional differences, astrocytes share a core of functions in the normal adult brain. Astrocytes help maintain brain function through direct neurotransmitter reuptake and recycling [18], maintenance of neuronal health and synapses via trophic support [19], synapse elimination directly [20] or indirectly through recruitment of microglia using the complement pathway [21], regulation of extracellular space and ion concentration such as potassium via the potassium inward rectifier 4.1 (Kir4.1/*KCNJ10*) [22,23], homeostatic maintenance of pH (i.e., via excitatory amino acid transporter (EAAT) regulation of glutamate), the maintenance of brain metabolism through the lactate shuttle, and regulation of local extracellular volume via the interactions of aquaporin-4 (AQP4) and Kir4.1 [24]. Astrocytes also exert more direct effect on neural function via gliotransmission, the release of neurotransmitters from non-neuronal cells [25], and through neurovascular coupling [26].

Similar to microglia, astrocytes are affected by aging, including their ability to maintain these basal functions [15]. In addition, several groups have demonstrated that, over multiple brain regions and ages, astrocytic neuroinflammatory genes are upregulated during normal aging [15,27], including genes specific to synapse elimination.

The functional alteration and increased reactivity in both astrocytes and microglia in normal aging is enhanced by brain insults such as in the context of neurodegenerative diseases. Enhanced microglial activation via the NF-κB pathway, or other proinflammatory pathways, can impact the overall neural architecture in several ways: by affecting neuronal function through direct signaling and through release of proinflammatory cytokines [28], by regulating the inflammatory state of non-neuronal cells that will in turn cause a variety of secondary effects in neurons [29], through modulation of extracellular proteins and protein aggregates [30,31,32], and through the direct inappropriate phagocytosis of neural and glial cells via the classical complement pathway [33,34]. Likewise, upon sensing an insult, astrocytes profoundly alter both their morphologies and transcriptomes. The classical activation state of astrocytes can be defined by an increase in the expression of glial fibrillary acidic protein (GFAP) or vimentin which coincides with thickening of the major processes resulting in cellular hypertrophy [35]. This was suggested to result in either a neurotoxic “A1” or neuroprotective “A2” phenotype, which can profoundly worsen or ameliorate the severity of the insult [29,36]. Importantly, like microglia, astrocytes in mouse models of neurodegeneration often have decreased expression of genes involved in their homeostatic roles, indicating reduction in neurosupportive functions [37,38,39,40]. For example, astrocytes in mouse models of Huntington’s disease have reduced expression of Kir4.1 which results in perturbed ion concentrations and increased excitability of neurons [10].

**Figure 1:**
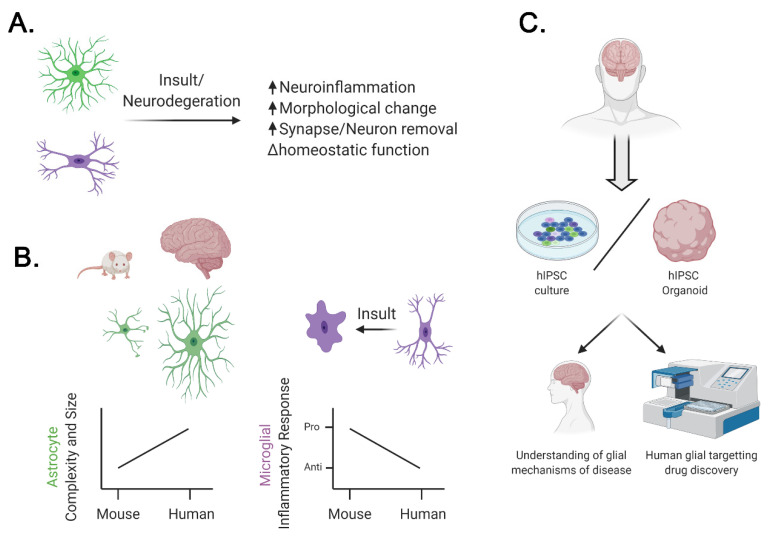
Bimodal role of glia in neurodegeneration and differences between human and mouse glia. (**A**) Both astrocytes and microglia are poised to be critical players during neural insult due to their close association and interactions with neurons. Thus, during a neurodegeneration, there is an interplay between reduction in homeostatic functions and participation in neuroinflammatory response. Loss of regular homeostatic functions of glia, including reduced regulation of ion and neurotransmitter concentration in the extracellular space as well as reduced secretion of neurotrophic factors, can promote disease pathogenesis, while compensatory increase in these functions can delay disease onset. Neuroinflammation in glia alters their morphology and increases secretion of pro- and anti- inflammatory cytokines and participation in removal of damaged synapses and neurons. While initially this may be beneficial, chronic neuroinflammation seems to be harmful. (**B**) Humans and mice differ in the complexity of astrocyte processes, which is increased in humans relative to mice (left), and in microglial neuroinflammatory response to secreted molecules, which is decreased in humans (right). (**C**) Patient-derived human-induced pluripotent stem cells (iPSCs) can be used to study the cellular and molecular processes contributing to glial changes in neurodegenerative diseases as well as for high-throughput testing of potential drugs. Created with BioRender.com.

Both astrocytes and microglia are poised to be critical players during neural insult due to their ability to sense the local neural environment, actively alter the cytokine load and overall neuroinflammatory state, and ability to maintain and restore homeostasis (Figure 1A). There are many studies examining the roles of astrocytes and microglia in animal models of neurodegenerative disease [41,42]. Some of those studies provide evidence that astrocytes and microglia are harmful and exacerbate neurodegeneration [41,43,44,45,46,47]. For example, reducing neuroinflammatory NF-κB signaling in astrocytes ameliorated synapse loss and cognitive impairments in mouse models of Alzheimer’s disease (AD) [48,49]. Similarly, restoring gamma frequency oscillations in the cortex of AD model mice increased phagocytic activity of microglia, reduced amyloid plaque load, and improved cognition [50]. There are also studies suggesting that glia can be neuroprotective, especially during the early stages of neurodegeneration [42,51,52]. For example, in early stages of disease progression, inhibiting astrogliosis exacerbated disease severity in the mouse model of spinocerebellar ataxia type 1 [52].

While these studies implicated loss of homeostatic, supportive functions and gain of neurotoxic functions as main glial contributions to neuronal dysfunction and death in mouse models of neurodegenerative diseases, the implications for human disease are not as straightforward.

## 2. Differences between Human and Mouse Glial Cells

The majority of research on the role of glia in human neurodegenerative diseases utilizes mouse models. As a consequence, most of our understanding applies to mouse glia. Many aspects of glial biology are conserved across species. However, divergence between species can have direct effects on glial gene expression and morphology. Moreover, given the role of glia in managing and responding to the cellular environment, species differences can be amplified by small changes in the local environment of the central nervous system (CNS), including neuronal transcriptomics and lipidome and proteome expression. Together, these considerations caution researchers against straightforward extrapolation of mouse research to human conditions. Species differences in glia may contribute to the failure of many promising treatments from animal research in human clinical trials. The apparent differences between the human and murine glia stress the importance of increasing our understanding of comparative glial physiology and pathophysiology for clearer knowledge of human glia, more informed interpretation of mouse data, and improved development of successful therapies for human diseases.

Due to the relative paucity of tools to study them, the earliest reports of astrocyte species differences came from imaging of brain slices. Compared to mouse brains, human astrocytes are larger, have increased astrocyte to neuron ratio, have more complex cellular subtypes, and have more processes which cover more synapses (Figure 1B, Table S1). Whether this imparts novel functions assigned to astrocytes in the human cortex remains to be further investigated.

The human cerebral cortex has a glia-to-neuron ratio of 1.4 [53,54], whereas the rodent cerebral cortex has a glia-to-neuron ratio of just 0.4 [55]. When considering astrocytes alone, this means that astrocytes make up about 40% of the human cortex but only 25% of the mouse cortex [24,56].

In addition, human brains contain two additional specialized types of astrocytes not found in mice, such as varicose projection astrocytes and interlaminar astrocytes [57]. Interlaminar astrocytes, with cell bodies found in cortical layer I, have few projections that terminate in cortical layers II-III and are found in higher primates, including Old World monkeys. In contrast, varicose projection astrocytes, which have several long, unbranched processes, seem to be adult human specific. These long processes may play a role in long-distance intracortical communication and coordination of neuronal activities, potentially contributing to the development of cognitive functions that are specific to higher primates and humans.

Even the astrocyte subtypes present in both species, such as fibrous and protoplasmic astrocytes, exhibit differences, with those in the human cortex exhibiting greater size and complexity. For instance, protoplasmic astrocytes are the most abundant type of astrocyte present in both humans and rodents [58]. Their processes cover blood vessels, neuronal cell bodies, and numerous synapses, allowing them to coordinate blood flow and neurotransmitter release in response to changes in synaptic activity [59]. However, protoplasmic astrocytes in the human cortex are 2.6-fold larger in diameter and extend 10-fold more GFAP-positive primary processes than those in the mouse cortex [60]. This allows as many as two million synapses to be supported within the domain of a single human astrocyte, whereas a rodent astrocyte domain covers about 90,000 synapses. In addition to this increase in synaptic connections, human protoplasmic astrocytes also have more rapid responses to environmental changes within their domain. Indeed, human protoplasmic astrocytes from acutely resected surgical tissue have been shown to propagate Ca^2+^ waves approximately fourfold faster than rodents [57].

As one might expect, given these notable morphological differences, there are significant molecular differences between human and mouse astrocytes. Unbiased genome-wide comparison of the human and mouse astrocytes revealed over 600 genes enriched in human but not in mouse astrocytes. Some of these genes may explain differences in functional properties between human and mouse astrocytes. For instance, one of the human astrocyte-specific genes is ryanodine receptor 3 (*RYR3*), a calcium permeable ion channel that regulates release of calcium from the endoplasmic reticulum (ER). This may contribute to increased calcium propagation of human astrocytes compared to mouse astrocytes [61,62].

It is important to note that, despite the above differences, there is significant overlap in genes expressed both in human and mouse astrocytes. Such conserved gene expression changes include classic astrocyte-specific genes such as *GFAP*, *ALDH1L1*, *GLUL*, *AQP4*, *SLC1A2*, and *SLC1A3* [61]. In addition, astrocytes in many human neurodegenerative diseases, including ALS, AD, Huntington’s disease, and Parkinson’s disease exhibit expression of *C3*, which has been identified as one of the core genes in mouse neurotoxic microglia-activated astrocytes [36,63].

Research is ongoing as to the role of these differences in the diversity, complexity, and functional properties of human and rodent astrocytes in physiological and pathological conditions. Current hypotheses presuppose that, in healthy brain, these functional differences may contribute to differences in cognitive ability. Indeed, recent work suggests that engrafting human glial progenitor cells (GPCs) into neonatal immunodeficient mice enhanced the cognitive ability of the chimeric host mice [64]. In adulthood, the recipient mouse brains showed a high number of human GPCs and human astrocytes which were gap junction coupled to host astroglia. Importantly, the engrafted human astrocytes retained the larger size and increased complexity as well as the enhanced propagation of Ca^2+^ signals characteristic of human astrocytes. Recipient chimeric mice exhibited both enhanced long-term potentiation and learning relative to mouse GPC allograft controls [61]. Together, these findings suggest that human astrocytes may contribute to an increase in computational power of the human cortex. While these unique functions of human astrocytes may be advantageous in physiological conditions, it is reasonable to propose that any pathological changes in human astrocytes could have a worse and wider reaching consequence than in mouse astrocytes.

Microglia are commonly known as the immune cells of the brain. As the immune system is a prime target for evolutionary change, caused by evolving pathogens driving divergence between different species [65], it is not surprising that there are significant differences between mouse and human microglia. Divergence in human and mouse microglial features may in part account for differences in disease progression and drug treatment efficacy between mouse models and human trials.

While human and mouse microglia share basic similarities such as expression of the intracellular calcium-binding protein induction of brown adipocytes 1 (Iba-1) and transcription factor PU.1 [66,67,68], many interspecies differences have already been identified. Comparisons between human and mouse microglia in vitro have revealed some interesting differences, such as the increased capacity of mouse microglia to proliferate in vitro relative to human microglia [69,70].

The two species seemingly differ in the ways that microglia modulate neuroinflammation in response to secreted molecules (Figure 1B, Table S1). The well-described response of mouse microglia to transforming growth factor-β1 (TGFβ1) is not seen in cultured human microglia. The T-cell cytokine interferon-γ (IFNγ) has been shown to increase expression of class II major histocompatibility complex (MHCII) in mice and human leukocyte antigen (HLA) in human microglia. Interestingly, TGFβ1 blocks this IFNγ-induced response in mouse microglia [71] but not in human microglia [72]. This is important because increased HLA and MHCII expression are associated with neuroinflammatory response to brain injury and disease in both mouse models and human post-mortem brain tissue [73,74,75].

Human and mouse microglia also react differently to colony-stimulating factor (CSF) signals. When presented with granulocyte–macrophage or macrophage (GM-CSF or M-CSF), cultured mouse microglial cells upregulate MHCII, increase their release of proinflammatory cytokines, and take on a reactive morphology [76,77]. The same stimuli have the opposite effect in human microglia, causing them to reduce MHCII expression and increase production of anti-inflammatory cytokine interleukin-10 (IL-10) [78].

Like with astrocytes, weighted gene co-expression network analysis on microarrays from human and mouse brains have revealed similarities and differences in the transcriptomes of the two species’ microglia [79,80,81]. While modules of highly co-expressed genes identified in both mouse and human brains were mostly strongly preserved, microglial modules differed in their correlation to markers of dementia and Alzheimer’s disease (AD) progression. Mouse microglial modules contained very few of these disease genes, whereas these genes were highly represented in human microglial modules. Additionally, the researchers measured the overlap between each module and AD related genes [82], finding that only human microglial modules showed significant enrichment for AD genes. These findings provide important insight to the difference between human and murine microglia in AD and neurodegeneration. The enrichment of dementia and AD genes in microglia may in part underlie neuroinflammation in human brain disease and suggest a limitation of the utility of mouse models in the study of AD and other neurodegenerative diseases. Thus, it is important that researchers keep in mind these interspecies differences as well as the observation that neuronal dysfunction is only one of many pathological processes contributing to the progression of AD.

## 3. Contribution of Glial Cells to Human Neurodegenerative Diseases

Genetic analysis, epidemiological studies, gene expression, and imaging in human patients demonstrate the contribution of glial cell dysfunction to neurodegeneration (Table S2). Genetic analysis using genome-wide associated studies identified a link between immune response and risk for AD [83]. Additionally, epidemiological studies linking the use of anti-inflammatory drugs with a decreased risk for dementia suggested that inflammation may be a contributing factor [84].

Pathological analysis of late onset AD (LOAD) with the integrative network-based approach highlighted immune- and microglia-specific modules for relevance to pathology [85]. Intriguingly, determination of phenotypic traits that are linked to human resilience to Alzheimer’s pathology identified activated glia as likely mediators of neurotoxicity and altered cognition. For example, quantitative histopathological and biochemical comparison of post-mortem brains from cognitively intact, AD-resilient, controls, and individuals with AD-associated dementia demonstrated several significant differences, including markers of gliosis [86]. AD-resilient individuals exhibited decreased activation of astrocytes and microglia, decreased production of proinflammatory cytokines, and increased production of neurotrophic factors [87]. This suggests that reduced neuroinflammation may be protective to AD pathology.

Indeed, disruption of normal astrocytic functions may also contribute to AD pathology. Analysis of gene expression changes in patients indicates that AD is associated with altered immune response and mitochondrial processes in astrocytes [88]. The percentage of senescent astrocytes, marked by p16 expression, increases with aging and is further increased in Alzheimer’s disease [89]. Senescent astrocytes have decreased expression of glutamate transporters that may disrupt the normal astrocytic uptake and potentially lead to the accumulation of excitotoxic extracellular glutamate [90]. Additionally, a number of astrocytic senescence associated secretory phenotype (SASP) genes appear to promote the formation of hyperphosphorylated tau, which in turn leads to neurofibrillary tangle (NFT) formation, neuronal dysfunction, and cognitive decline [90]. Similarly, in patients with Parkinson’s disease (PD), accumulations of α-synuclein and Lewy body pathology in the substantia nigra pars compacta neurons may in part be a result of elevated SASP-induced cytokines, such as IL-1β, IL-6, and TNFα, generated by senescent astrocytes and other cells [90].

Human aging has a stronger effect on gene expression changes in astrocytes and microglia than in other brain cells, to the extent that human astrocytes and microglia are much better at predicting the age than oligodendrocytes and neurons [91]. This may indicate that aging, one of the most important risk factors in many neurodegenerative diseases, contributes to neurodegenerative changes in neurons through altered glial function. Specifically, while microglial gene expression demonstrates increased immune response throughout the brain, astrocyte-specific genes show region-specific changes with aging, with most pronounced changes seen in hippocampus and substantia nigra [91].

In Huntington’s disease (HD), both human plasma and cerebrospinal fluid (CSF) had increased proinflammatory mediators, such as cytokines interleukin-6 (IL-6) and interleukin-8 (IL-8). This indicates that immune activation is occurring in both in periphery and in CNS. Interestingly, not only is this increase in IL-6 levels in plasma detectable in HD gene carriers 16 years prior to predicted onset of symptoms [92], but these levels also correlate well with clinical severity scores in pre-manifest and manifest HD gene carriers. Current hypotheses suggest that these plasma and CSF changes are directly caused by the expression of mutant huntingtin (HTT) protein in monocytes and microglia, respectively.

Similar to astrocytes, human microglia have been shown to undergo dystrophic changes with normal aging and in Alzheimer’s brains. For instance, gray matter microglia have significantly reduced total arborization area as evidenced by their shorter process lengths and reduced branching that was exacerbated in AD brains as compared to age-matched control brains [93].

In addition, AD microglia associated with amyloid beta (Aβ) plaques demonstrate downregulation of genes involved in their normal function, including *P2Y12*, *P2Y13*, *CX3CR1*, *CD33* and *TMEM119*; reduced mobility; phagocytic activity [94]; and decreased barrier function [32], while simultaneously upregulating genes that contribute to synaptic loss [34,95,96]. This has led to a prevailing hypothesis that decrease in microglial homeostatic functions contributes to AD pathogenesis.

## 4. Modeling the Role of Glia in Neurodegenerative Disease Using Human iPSCs

The field of neurodegeneration has recently turned to the use of human-induced pluripotent stem cells and similar in vitro models to study the cellular and molecular processes that may be regulating disease in living cells. Indeed, to best model the biochemical signature of specific diseases, human glial cells can be differentiated from the stem cells derived from a patient’s own adult somatic cells. In the early 2000’s, Yamanaka and Takahashi reported the first reprogramming of adult somatic cells to a pluripotent, stem cell-like state [97]. Transfection with factors that drive proliferation and pluripotency, usually a combination of *Klf4*, *Oct3/4*, *Sox2*, and *c-Myc*, transforms cells isolated from patient’s skin, urine, or blood [97,98,99,100] to a stem-cell like state. Known as induced pluripotent stem cells (iPSCs), these cells can then be differentiated into specific cell types of interest like astrocytes [101] or microglia [102] via transfection with cell-type-specific transcription factors or by the application of environmental signals that mimic development. The clear advantage of this model is the human origin and maintenance of the donor’s genome, which allows for the recapitulation of the complex genetic features that may contribute to a disease. This is particularly important when considering neurodegenerative diseases such as AD and PD, where most animal models rely on single gene mutations despite sporadic cases making up the majority of the patient population. Even across single gene mutations in a neurodegenerative disease, such as early onset AD, evidence shows that cell-type-specific molecular pathology differs [103]. When considering translational models for therapeutic assessment, a human cell-type-specific disease model could serve as a useful translational bridge between mice and patients.

Human iPSC-derived astrocytes demonstrate mature transcriptional expression, morphology, calcium response, and immune reactivity [98,101,104]. In addition to these general astrocyte characteristics, recent publications have suggested that human iPSC-derived astrocytes may be directed to take on regionally distinct molecular profiles [105]. This suggests that human iPSC-derived astrocytes can be used to model regionally specific aspects of neurodegenerative diseases, such as AD, PD, and ALS. Astrocytes derived from patients with neurodegenerative diseases exhibit molecular changes that affect cell survival and function, mirroring changes seen in human post-mortem tissue and mouse models. A 2017 paper published by Chinta and colleagues shows that the pesticide paraquat, known to increase the risk for PD in patients, correlates with increased cellular senescence in human post-mortem tissue taken from the substantia nigra pars compacta. Importantly, this effect was recapitulated in human iPSC-derived astrocytes, which demonstrated a marked decrease in proliferation and increased DNA damage signaling after application of paraquat in vitro [106].

Similarly, increased vacuolization, which has been noted as a disease-associated phenotype in leukocytes, can be observed in HD patient iPSC-derived astrocytes in a repeat expansion dependent manner [107]. The ALS-associated superoxide dismutase 1 (*SOD1*) mutation specifically dysregulates the neuroprotective EphB1-ephrin-B1-STAT3 signaling pathway in human iPSC-derived astrocytes, while leaving the IL-6 responsive activity of STAT3 intact. This finding indicates a molecular pathway that imparts increased risk for ALS through an astrocytic cell autonomous mechanism [108,109]. Uncovering molecular mechanisms of disease pathogenesis in glial cells like this both broadens our understanding of the non-cell autonomous contributions to neural degeneration and reveals key glial vulnerable pathways that may be targeted to delay disease onset (Figure 1C).

Microglia made from human iPSCs demonstrate characteristic phagocytic activity, immune response, morphology, and marker expression, the last of which being notably enhanced through co-culture with neurons [102]. Altered phagocytosis is one of the most common functional changes noted in patient iPSC-derived microglia. Phagocytosis is important for the efficient clearance of oligomers, synapses, and damaged cells from the CNS. Alpha synuclein (*SNCA*) gene triplication causes a marked increase in intracellular alpha-synuclein expression in iPSC-derived macrophages, contributing to their reduced phagocytic functionality [110]. Mutation in leucine-rich repeat kinase 2 (*LRRK2*) confers increased risk for PD to patients. The mechanism responsible is still under investigation, but recent work has shown that LRRK2 expression is strongly upregulated in human iPSC-derived microglia following interferon γ (IFNγ) treatment and results in LRRK2 recruitment to maturing phagosomes [111]. The expression of cholesterol carrier apolipoprotein E4 (APOE4) and reduced function of the immune receptor triggering receptor expressed on myeloid cells (TREM2) are both associated with a higher risk for developing AD. While APOE4 expression seems to boost immune reactivity of microglia, differential gene expression and morphological analysis demonstrate a clear reduction in ramification and movement, which may underlie observed slower phagocytic clearance [103,112]. TREM2 missense expression appears to cause deficits specifically in the phagocytosis of apoptotic cells [113]. In keeping with this, TREM2 loss of function exhibits largely normal debris clearance but deficits in cholesterol metabolism, ultimately leading to toxicity and immune activation through the formation of lipid aggregates [114] This could suggest a broader mechanism whereby poor lipid metabolism and clearance contributes to the underlying pathologic mechanism of late stage AD. Notably, *presenilin 1* (*PSEN1*) and familial AD risk genes act through an apparently different mechanism, decreasing microglial cytokine production and increasing process motility without altering phagocytic function [103].

Co-culture of patient iPSC-derived astrocytes and microglia with neurons can be used to further unravel the non-cell autonomous effects of glial dysfunction in neurodegenerative disease. The AD-associated *PSEN1 ΔE9* mutation drastically alters human iPSC-derived astrocyte function, causing aberrant calcium signaling, reactive oxygen species (ROS) production, and cytokine secretion. When co-cultured with healthy neurons, these AD astrocytes contribute to reduced neuronal activity [115]. Neuronal health and synapse maintenance are affected by the expression of the cholesterol carrier APOE4, which is highly correlated with a patient’s risk for AD. Human iPSC-derived neurons grown in serum from human APOE 4/4 astrocytes show reduced synapse expression and survival compared to those grown in human APOE 3/3 astrocyte serum [116]. Together, these studies demonstrate the power of co-culture for gaining insight into the role of glial cells in the early cellular pathogenesis of AD. Similar effects have been unraveled for a number of neurodegenerative diseases, including ALS [109,117,118], PD [119], and HD [120]. Microglia add an additional dimension to neurodegenerative disease modeling in vitro and can be incorporated into embryoid bodies or other 3D culturing methods to create combined systems. Indeed, co-culture with neurons appears to improve the maturation and function of iPSC-derived microglia [121]. A tri-culture system was recently described in a paper from Park and colleagues who reported recapitulation of AD pathology and proinflammatory activation in a 3D culture containing iPSC-derived neurons, astrocytes, and microglia [122]. Such a model could provide rich insights into the cellular and molecular pathology of various neurodegenerative diseases in which glia play a large role (Figure 1C).

While there are many advantages, researchers must be aware that iPSC creation requires the erasure of established epigenetic profiles in cells, returning cells to an embryonic-like age. Recent work suggests that this may be avoided through direct transduction of somatic cells into neurons, which maintains the epigenetic age [123]. Additionally, human iPSC-derived neurons, astrocytes, and microglia may be aged beyond what is possible in vitro by implanting differentiated cells into mice to create chimeras [107,124]. These can then be used to study the effects of specific affected human cell types on disease-associated phenotype expression in vivo. Indeed, astrocytes derived from human glial progenitor cells implanted in mice have been shown to retain human cell characteristics [64]. Interestingly, human iPSC-derived microglia chimeras exhibit tiling, regionally heterogeneous gene expression, and normal response to insults both chronic and acute [125,126].

Despite these limitations, astrocytes and microglia derived from patients already provided evidence supporting the concept that perturbations in glial housekeeping and defense functions contribute to disease and are poised to increase our understanding of pathogenesis of neurodegenerative disease and high-throughput drug testing in the future.

In conclusion, even though rodent models have enlightened many aspects of the function of glia, we urge some caution when using rodents as a translational model due to the differences between mouse and human glia. Specifically, the increases in complexity, glia-to-neuron ratio and density, sub-differentiation of higher primate and human glia, large-scale transcriptomic differences, as well as reactivity should be accounted for when studying rodent astrocytes and microglia. Due to these factors, we recommend combinatorial rodent and human iPSC studies in order to obtain more in-depth understandings of the role of glia in neurodegenerative disease to increase the impact of glial translational research.

## Supplementary Materials

Supplementary materials can be found at https://www.mdpi.com/1422-0067/21/23/9188/s1.
