# Supplementary material for: Glia in Neurodegeneration: The Housekeeper, the Defender and the Perpetrator
Source: Int J Mol Sci. 2020 Dec 2;21(23):9188. doi: 10.3390/ijms21239188 (PMC7730416; doi:10.3390/ijms21239188)
Supplement: Supplementary file 1 [file ijms-21-09188-s001.pdf]

**Table S1**

**Main differences between human and mouse glial cells**

| | **Human** | **Mouse** | **References** |
|---|---|---|---|
| **Astrocytes** | greater morphological complexity (i.e. longer and more branched processes) | less morphological complexity | [58][64] |
| | ~ 40% of brain cells | ~ 25% of brain cells | [24][56] |
| | varicose projection, intralaminar, fibrous, protoplasmic | fibrous, protoplasmic | [57][58] |
| | large GFAP+ protoplasmic astrocytes can cover up to $2X10^6$ synapses | relatively smaller protoplasmic astrocytes covering up to $9X10^4$ synapses | [60] |
| | rapidly responding protoplasmic astrocytes | slower response in protoplasmic astrocytes | [57] |
| | more genes enriched in human astrocytes | fewer genes enriched in human astrocytes | [61] |
| | increased Ca++ propagation relative to mouse astrocytes | reduced Ca++ propagation relative to human astrocytes | [61][62] |
| | express GFAP, ALDH1L1, GLUL, AQP4, SLC1A2, SLC1A3 (also C3 in neurodegeneration) | express GFAP, ALDH1L1, GLUL, AQP4, SLC1A2, and SLC1A3 | [61][63] |
| | | | |
| | **Human** | **Mouse** | **References** |
| **Microglia** | express Iba1, PU.1 | express Iba1, PU.1 | [66][67][68] |
| | poor proliferation in vitro | readily proliferate in vitro | [69][70] |
| | TGFβ1 does not block IFNγ-induced increase in HLA | TGFβ1 blocks IFNγ-induced increase in MHCII | [71][72] |
| | GM-CSF or M-CSF decrease MHCII and anti-inflammatory IL-10 release | GM-CSF or M-CSF increase MHCII and proinflammatory cytokine release | [76][77][78] |
| | multiple genes expressed in neurodegeneration (e.g. AD related) | no AD -specific genes expressed in microglia | [82] |

**Table S2**

**Involvement of glial cells in human neurodegenerative diseases**

| **Neurodegeneration (e.g. late onset AD, PD, HD, etc)** | **References** |
|---|---|
| altered immune response and mitochondrial function in AD astrocytes | [88] |
| larger increase in p16 expression in AD astrocytes | [89] |
| decreased astrocyte glutamate uptake, increased SASP genes & hyperphosphorylated tau in cognitive decline and PD | [90] |
| increased microglial activation with elevated brain-wide immune response, while astrocyte activation in neurodegeneration in hippocampus & substantia nigra | [91] |
| increased microglial IL-6 in HD | [92] |
| decreased microglial arbor ramification in AD gray matter vs normal brain | [93] |
| microglia associated with amyloid beta plaques have down-regulated normally functioning genes, P2Y12, P2Y13, CX3CR1, CD33 and TMEM119; reduced mobility; phagocytic activity; decreased barrier function and increased gene expression related synapse loss | [94][95][96] [97] |
